# Differences in rhizosphere microbial communities between *Fusarium* wilt-resistant and susceptible watermelon cultivars

**DOI:** 10.1128/spectrum.04149-25

**Published:** 2026-08-10

**Authors:** Lulu Qiu, Jinyan Huang, Guifen Li, Yi He, Zhiyang Wei, Shangdong Yang

**Affiliations:** 1 National Experimental Teaching Demonstration Center of Plant Science, Guangxi Key Laboratory of Agricultural Products Safety, Agricultural College, Guangxi University 12664, Nanning, Guangxi, China; 2 Horticulture Research Institute, Guangxi Academy of Agricultural Sciences, Nanning, Guangxi, China; Instituto de Ecología, A.C. (INECOL), Pátzcuaro, Michoacán, Mexico

**Keywords:** *Citrullus lanatus*, plant–microbe interactions, genotype, high-throughput sequencing, microbiome

## Abstract

To elucidate genotype-associated differences in rhizosphere microbial community assembly, this study compared the microbiomes of three *Fusarium* wilt-resistant and three susceptible watermelon cultivars using amplicon sequencing. Results revealed distinct bacterial and fungal community structures between the two groups. Notably, resistant cultivars harbored a higher number of unique operational taxonomic units and displayed greater fungal richness compared to their susceptible counterparts. Beyond taxonomic composition, co-occurrence network analysis demonstrated that the fungal community within the resistant group exhibited a more highly connected network topology. Additionally, functional prediction highlighted significant divergence in potential functional profiles, including variations in *Forms_Biofilms* and *Contains_Mobile_Elements*. Collectively, these findings demonstrate that rhizosphere microbial composition, diversity, and network complexity are closely linked to watermelon resistance phenotypes. This comprehensive characterization of genotype-driven microbiome variation offers a critical basis for understanding plant–microbe interactions and their potential to enhance plant health.

**IMPORTANCE:**

*Fusarium* wilt is one of the most destructive diseases affecting watermelon production worldwide, yet the role of soil microbes in helping plants resist this disease has remained unclear. This study shows that disease-related;resistant watermelon plants naturally recruit a richer and more cooperative community of beneficial microbes around their roots. These microbes may help protect the plant by improving nutrient use, forming biofilms that enhance microbial stability, and competing with or inhibiting harmful pathogens. In contrast, susceptible plants rely on only a few protective microbes, making their root environment less stable and more vulnerable to infection. By revealing how plant genetics shape the assembly and function of root-associated microbial communities, this work provides a scientific foundation for developing microbiome-based strategies-such as microbial inoculants or breeding for microbiome‑friendly cultivars-to improve crop resilience and reduce reliance on chemical pesticides.

## INTRODUCTION

Soil microbial communities are integral components of ecosystems, with the rhizosphere microbiome serving as a key ecological determinant of host plant health and productivity ( 1, 2). As the microenvironment where root–soil interactions are most intense, rhizosphere microbiome assembly is shaped by multiple selective pressures, including host genotype, root exudate composition, soil physicochemical properties, and local habitat disturbances (3, 2). Among these factors, plant genotype is considered a primary endogenous driver of community differentiation. Distinct genotypes regulate carbon allocation, secondary metabolite secretion profiles, root architecture, and developmental dynamics (4, 5), thereby altering microbial colonization thresholds and modulating resource availability and ultimately shaping stable, genotype-specific rhizosphere microbiome assemblages (6–8). This process reflects deterministic assembly in community ecology. Coupled with priority effects and niche construction, it facilitates the recruitment and assembly of core microbial taxa (9, 10).

Watermelon (*Citrullus lanatus*) is highly valued worldwide for its exceptional nutritional content and flavor and is cultivated extensively across the globe. According to statistics from the Food and Agriculture Organization of the United Nations (FAO, 2023; accessed via FAOSTAT database [https://www.fao.org/faostat/] on 1 March 2025), the total global watermelon production exceeded 100 million tons. However, large-scale watermelon cultivation is under serious threat from *Fusarium* wilt, caused by *Fusarium oxysporum f. sp. niveum*, a soil-borne fungal pathogen specifically infecting watermelon. Studies have shown significant variation in soil microbial community structures in *Fusarium* wilt resistance among different watermelon cultivars (11). This variation in disease resistance may be closely linked to the composition and function of rhizosphere microbial communities. Although the genotype–microbiome–phenotype relationship has been demonstrated across multiple crop systems (12), the ecological basis of rhizosphere microbiome differences between *Fusarium* wilt-resistant and -susceptible watermelon cultivars remains unclear. However, efforts to explain these microbiome differences have often focused on single strains or isolated mechanisms (such as pathogen antagonism, nutrient competition, and induced systemic resistance), potentially overlooking the fact that microbial community functions as synergistic networks with complementary roles (13, 14). Studies of plant-associated microbiomes have highlighted that microbial community composition, diversity, and co-occurrence patterns can vary substantially among host genotypes and may influence microbiome assembly processes (10, 15, 16). However, the extent to which host genetic variation shapes rhizosphere microbial community structure across different plant systems remains incompletely understood.

To address this gap, this study aimed to decipher the link between genotype-driven rhizosphere assembly and *Fusarium* wilt resistance in watermelon. Three hypotheses were tested: (i) resistant and susceptible genotypes recruit distinct microbiomes, characterized by divergence in diversity, composition, and core taxa; (ii) these genotypes harbor microbial networks with distinct topologies, indicative of differential community organization; and (iii) genotype-specific functional profiles are associated with shifts in community structure.

Accordingly, this study conducts a comparative analysis of the rhizosphere microbial community composition between *Fusarium* wilt-resistant and -susceptible watermelon cultivars. By linking structural characteristics with functional potential, the study aimed to elucidate the ecological basis of disease resistance and characterize the protective microbial assemblage in resistant plants, thereby advancing the understanding of microbiome-mediated plant defense.

## MATERIALS AND METHODS

### Field site description and experimental designs

The soil used in this study was collected from the same experimental field at the Horticultural Research Institute, Guangxi Academy of Agricultural Sciences (108º17′E, 22º51′N). The soil type was red soil with a pH of 4.54, organic matter content of 10.06 g/kg, available nitrogen of 91.5 mg/kg, available phosphorus of 17.3 mg/kg, available potassium of 80.5 mg/kg, total nitrogen of 0.83 g/kg, total phosphorus of 0.67 g/kg, and total potassium of 8.9 g/kg. The planting soil in the greenhouse was thoroughly mixed to minimize differences among sampling sites, and multiple plots were established for the randomized cultivation of watermelon varieties with different resistance to *Fusarium* wilt, thereby ensuring that the physicochemical properties and initial microbial communities of the soil were as consistent as possible. Six watermelon cultivars used in this study were provided by the watermelon breeding team of the Horticultural Research Institute, Guangxi Academy of Agricultural Sciences, including Gui Ziye 2, Gui Ziye 3, Guixuan 118, Guixiao Xiang, Guixiao Hei, and Guixiao Mei. These cultivars are self-bred lines developed by the same breeding program. Based on KASP molecular marker analysis (17), Gui Ziye 2, Gui Ziye 3, and Guixuan 118 were identified as *Fusarium* wilt resistant, whereas Guixiao Xiang, Guixiao Hei, and Guixiao Mei were classified as susceptible. None of these cultivars have been officially registered or commercialized, and no known genetic relationship exists among them. The experiment was conducted using a randomized complete block design with four replicates. Each replicate plot contained six plants of a single cultivar, resulting in a total of 24 plants per cultivar. All cultivars were managed uniformly following identical cultivation practices. Field operations (sowing, transplanting, and management) were performed simultaneously for all cultivars.

### Soil samples collection

Sowing was carried out on 6 March 2023. Rhizosphere soil samples from wilt-resistant and susceptible watermelon cultivars were randomly collected from each cultivar on 26 May 2023, following the method of Yang et al. (18) with modifications. Briefly, using a sterilized spade, soil containing the root system was excavated at a depth of approximately 30 cm and within a 40 cm diameter around the plant base. The entire plant was then carefully pulled up. Loose bulk soil was gently shaken off, and the remaining soil adhering closely to the roots (rhizosphere soil) was collected. The collected rhizosphere soil was placed into a labeled sterile sealed bag and transported to the laboratory in a box containing ice packs. Simultaneously, soil samples from adjacent areas not planted with watermelon were collected to serve as background samples (CK). All soil samples were sieved through a 2 mm stainless steel mesh and stored at 4°C for immediate analysis or at −80°C for subsequent analysis. A total of 21 soil samples were collected, including 18 rhizosphere samples (9 from resistant cultivars and 9 from susceptible cultivars) and 3 background soil samples. For each replicate, 2–3 plants were randomly selected, and their rhizosphere soils were pooled to generate one biological replicate.

### Test methods

### DNA extraction, PCR amplification, and sequencing

Total DNA was extracted from watermelon rhizosphere soil samples using the FastDNA Spin Kit (MP Biomedicals, Santa Ana, CA, USA). The concentration and purity of the extracted genomic DNA were assessed via 1% agarose gel electrophoresis. DNA concentration and purity were determined using a NanoDrop 2000 spectrophotometer (Thermo Scientific, Waltham, MA, USA).

Illumina MiSeq sequencing was performed as follows: PCR products from the same sample were recovered: PCR amplification was performed using TransStart FastPfu DNA Polymerase (TransGen Biotech, Beijing, China; Cat. No. AP221-02). The PCR conditions were optimized to ensure high fidelity and efficiency. Amplifications were conducted in an ABI GeneAmp 9700 thermocycler (Applied Biosystems, Foster City, CA, USA). The bacterial 16S rRNA gene was amplified using the primer pair 338F (5′-ACTCCTACGGGAGGCAGCAG-3′) and 806R (5′-GGACTACHVGGGTWTCTAAT-3′). For fungal ITS region amplification, the primer pair ITS1F (5′-CTTGGTCATTTAGAGGAAGTAA-3′) and ITS2R (5′-GCTGCGTTCTTCATCGATGC-3′) was used. The PCR system consisted of 4 μL of 5× FastPfu Buffer, 2 μL of 2.5 mM dNTPs, 0.8 μL of forward primer (5 μM), 0.8 μL of reverse primer (5 μM), 0.4 μL of FastPfu Polymerase, 0.2 μL of BSA, and 10 ng of template DNA, with ddH_2_O added to a final volume of 20 μL. The amplification program was as follows: initial denaturation at 95°C for 3 min, followed by cycling (27 cycles for the 16S rRNA gene; 35 cycles for the ITS gene), each cycle consisting of denaturation at 95 °C for 30 s, annealing at 55°C for 30 s, and extension at 72°C for 45 s. This was followed by a final extension at 72°C for 10 min, and the reaction was terminated at 4°C. Each sample was analyzed in triplicate. The PCR products from the same sample were pooled and detected using 2% agarose gel electrophoresis. Gel slices containing the target bands were excised and purified using the AxyPrep DNA Gel Extraction Kit (Axygen, Union City, CA, USA), followed by elution with Tris-HCl buffer. The purified products were verified through 2% agarose gel electrophoresis. The PCR products were subsequently quantified using the QuantiFluor-ST Blue Fluorescence Quantification System (Promega, Madison, WI, USA) following the manufacturer’s instructions. The Illumina library construction was performed by Shanghai Majorbio Bio-Pharm Technology Co., Ltd. (Shanghai, China).

Paired-end raw sequencing reads were subjected to quality control using fastp (version 0.23.4; https://github.com/OpenGene/fastp), and read merging was performed with FLASH (version 1.2.11; http://www.cbcb.umd.edu/software/flash). Bases at the ends of reads with quality scores below 20 were trimmed using a 50 bp sliding window, and if the average quality score within the window fell below 20, bases downstream of the window were removed; reads shorter than 50 bp after quality control and those containing more than five ambiguous bases (*N*) were discarded. Paired-end reads were then merged into single sequences based on overlap relationships, with a minimum overlap length of 10 bp, and sequences with an overlap mismatch ratio greater than 0.2 were filtered out. Samples were distinguished and sequence orientation adjusted according to barcodes and primers at both ends of the sequences, with no mismatches allowed in barcodes and a maximum of two mismatches permitted in primer regions. The quality-controlled merged sequences were clustered into operational taxonomic units (OTUs) using USEARCH (version 11; http://drive5.com/usearch/) at a 97% sequence identity threshold, and chimeric sequences were removed. Taxonomic annotation of OTUs was performed using the RDP Classifier (version 2.11; https://github.com/rdpstaff/classifier) against the SILVA 138/16S_bacteria gene database with a confidence threshold of 70%, and sequences were further aligned to the 16S rRNA gene database (Release 138; http://www.arb-silva.de) and the fungal UNITE database (Release 8.0; http://unite.ut.ee/index.php). To minimize the impact of sequencing depth on downstream analyses, sample abundance data were rarefied (19).

### Statistical analyses

Statistical analyses were performed using Excel 2021 and SPSS 27.0. Data are presented as mean ± standard deviation (SD) to describe the distribution of alpha-diversity indices. Microbial alpha-diversity was assessed using the Shannon and Simpson indices (diversity) and the ACE and Chao1 indices (richness) calculated with mothur (http://www.mothur.org/wiki/Calculators). Partial least squares discriminant analysis (PLS-DA) using the “mixOmics” package in R (version 4.3.1), with results visualized using ggplot2. Discriminative taxa were identified using linear discriminant analysis effect size (LEfSe) on the platform (http://huttenhower.sph.harvard.edu/LEfSe), with linear discriminant analysis (LDA) scores used to determine taxa significantly contributing to group separation. Bacterial functional profiles were predicted using BugBase (https://bugbase.cs.umn.edu/index.html), and fungal functional guilds were assigned using FUNGuild (http://www.funguild.org/) based on taxonomic classification. Co-occurrence networks were constructed based on Spearman’s rank correlation coefficients, retaining only strong correlations (|*r*| > 0.6, *P* < 0.05). Networks were visualized in Gephi (version 0.10.1) using the Fruchterman–Reingold layout, and topological parameters including modularity, average path length, and centrality metrics were computed.

## RESULTS

### Soil microbial diversity in rhizosphere between *Fusarium* wilt-resistant and -susceptible watermelon cultivars

As shown in Table 1, the coverage obtained in this study exceeded 97%, indicating that the results reliably reflect the diversity and richness of soil bacterial and fungal communities. Statistical analysis revealed no significant differences in bacterial diversity and richness or fungal diversity between *Fusarium* wilt-resistant watermelon (KB) and susceptible watermelon (GB) cultivars (*P* > 0.05; Table 1). Notably, significant differences in fungal richness were observed among watermelon cultivars. The resistant cultivar Y2 exhibited the highest fungal richness (ACE and Chao1 indices) and was significantly higher than the susceptible cultivars Y10, Y11, and Y12. In contrast, the resistant cultivars Y3 and Y7 showed richness values comparable to some susceptible cultivars.

**TABLE 1:** *Alpha* diversity indexes of soil bacteria and fungi in rhizosphere between *Fusarium* wilt–resistant and susceptible watermelon cultivars^*a*^

Sample			Shannon	Simpson	Ace	Chao1	Coverage
Rhizosphere bacteria	CK		6.83 ± 0.01c	0.0044 ± 0.0002a	5,365.51 ± 103.82a	5,123.53 ± 70.01b	0.97
	GB	Y10	7.02 ± 0.02a	0.0037 ± 0.0001ab	6,113.61 ± 134.13a	5,762.23 ± 174.55a	0.97
		Y11	6.97 ± 0.02ab	0.0045 ± 0.0003a	5,770.90 ± 51.95b	5,527.92 ± 30.73a	0.97
		Y12	7.06 ± 0.07a	0.0036 ± 0.0006ab	5,958.12 ± 116.47ab	5,662.85 ± 128.91a	0.97
	KB	Y2	6.92 ± 0.11bc	0.0040 ± 0.0013ab	5,892.79 ± 327.50ab	5,539.17 ± 285.23a	0.97
		Y3	6.97 ± 0.01ab	0.0033 ± 0.0001b	5,970.97 ± 84.40ab	5,635.57 ± 65.34a	0.97
		Y7	7.03 ± 0.04a	0.0030 ± 0.0001b	5,836.21 ± 122.83ab	5,508.38 ± 173.44a	0.97
Rhizosphere fungi	CK		4.01 ± 0.08a	0.0511 ± 0.0065a	693.39 ± 34.73de	701.51 ± 46.10de	1.00
	GB	Y10	3.90 ± 0.26a	0.0618 ± 0.0093a	739.41 ± 57.44cd	738.01 ± 55.13cd	1.00
		Y11	4.04 ± 0.31a	0.0483 ± 0.0083a	648.82 ± 96.60e	647.75 ± 91.36e	1.00
		Y12	3.52 ± 0.30b	0.0862 ± 0.0146a	740.58 ± 63.12cd	734.50 ± 57.45cd	1.00
	KB	Y2	3.86 ± 0.26ab	0.0779 ± 0.0424a	864.04 ± 47.89a	852.72 ± 38.54a	1.00
		Y3	4.03 ± 0.25a	0.0561 ± 0.0099a	775.61 ± 35.26bc	784.12 ± 27.90bc	1.00
		Y7	3.69 ± 0.21ab	0.0784 ± 0.0210a	810.33 ± 23.63ab	808.78 ± 18.36ab	1.00

^*a*^ CK refers to the background soil; GB represents the *Fusarium* wilt-susceptible watermelon cultivar groups; KB represents the *Fusarium* wilt-resistant watermelon cultivar groups. Data in the table are means ± SD. Different lowercase letters in the same column indicate significant differences between different resistant groups of watermelon. *P* < 0.05.

**Fig 1:**
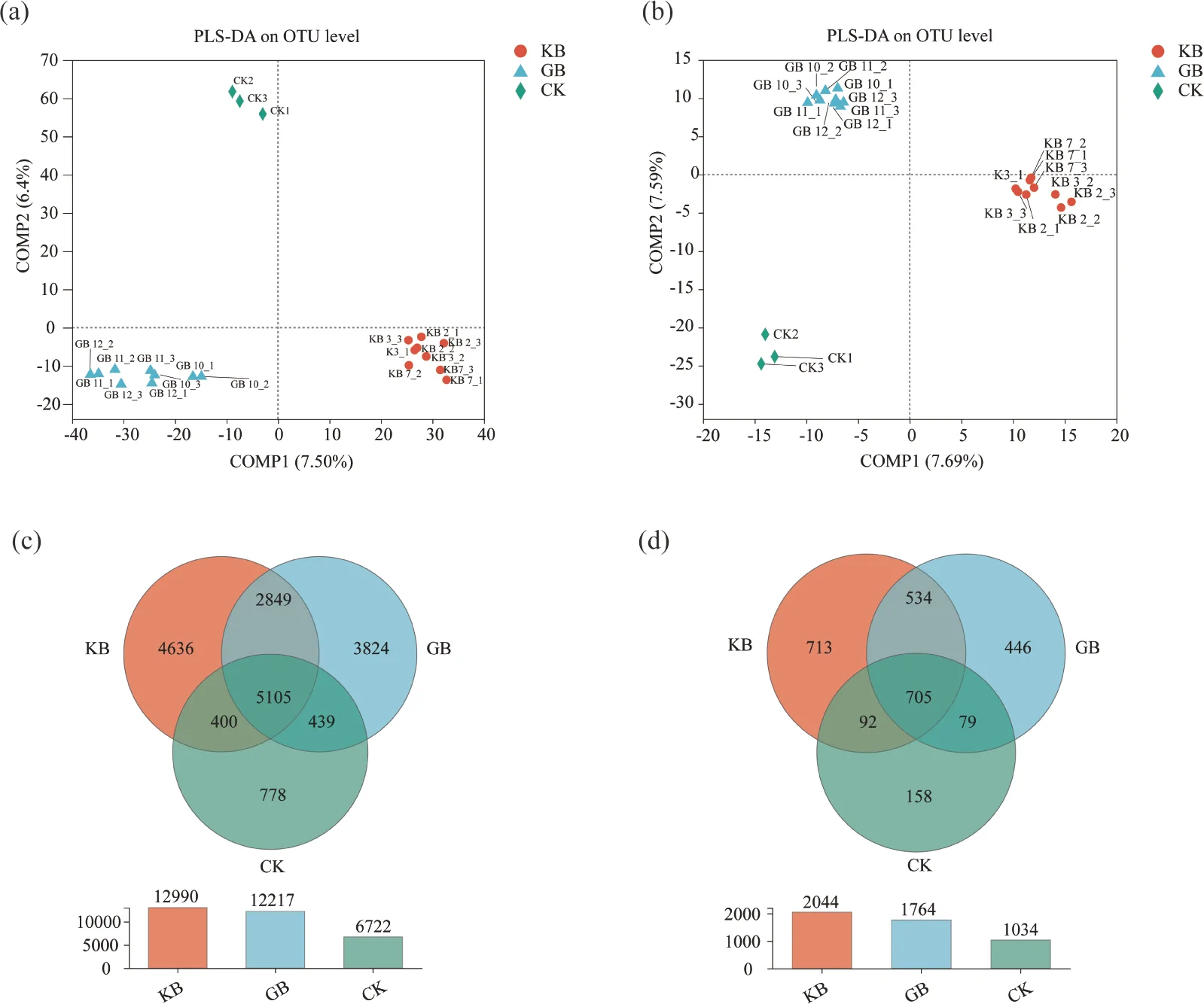
Structures of rhizosphere bacteria (**a**) and fungi (**b**) communities in different resistant watermelons. Venn diagram analyses of rhizosphere bacteria (**c**) and fungi (**d**) communities in different resistant watermelons. CK: background soil; GB: wilt-susceptible watermelon cultivar group; KB: wilt-resistant watermelon cultivar group.

PLS-DA at the OTU level revealed that rhizosphere bacterial (Fig. 1a) and fungal (Fig. 1b) communities of watermelon cultivars formed distinct clusters, indicating a strong association between *Fusarium* wilt resistance and rhizosphere microbiome assembly. Consistently, Venn diagram analysis also showed that the resistant cultivar group (KB) harbored more unique bacterial (Fig. 1c) and fungal (Fig. 1d) OTUs.

### Rhizosphere soil microbial community composition in *Fusarium* wilt-resistant and -susceptible watermelon cultivar groups

**Fig 2:**
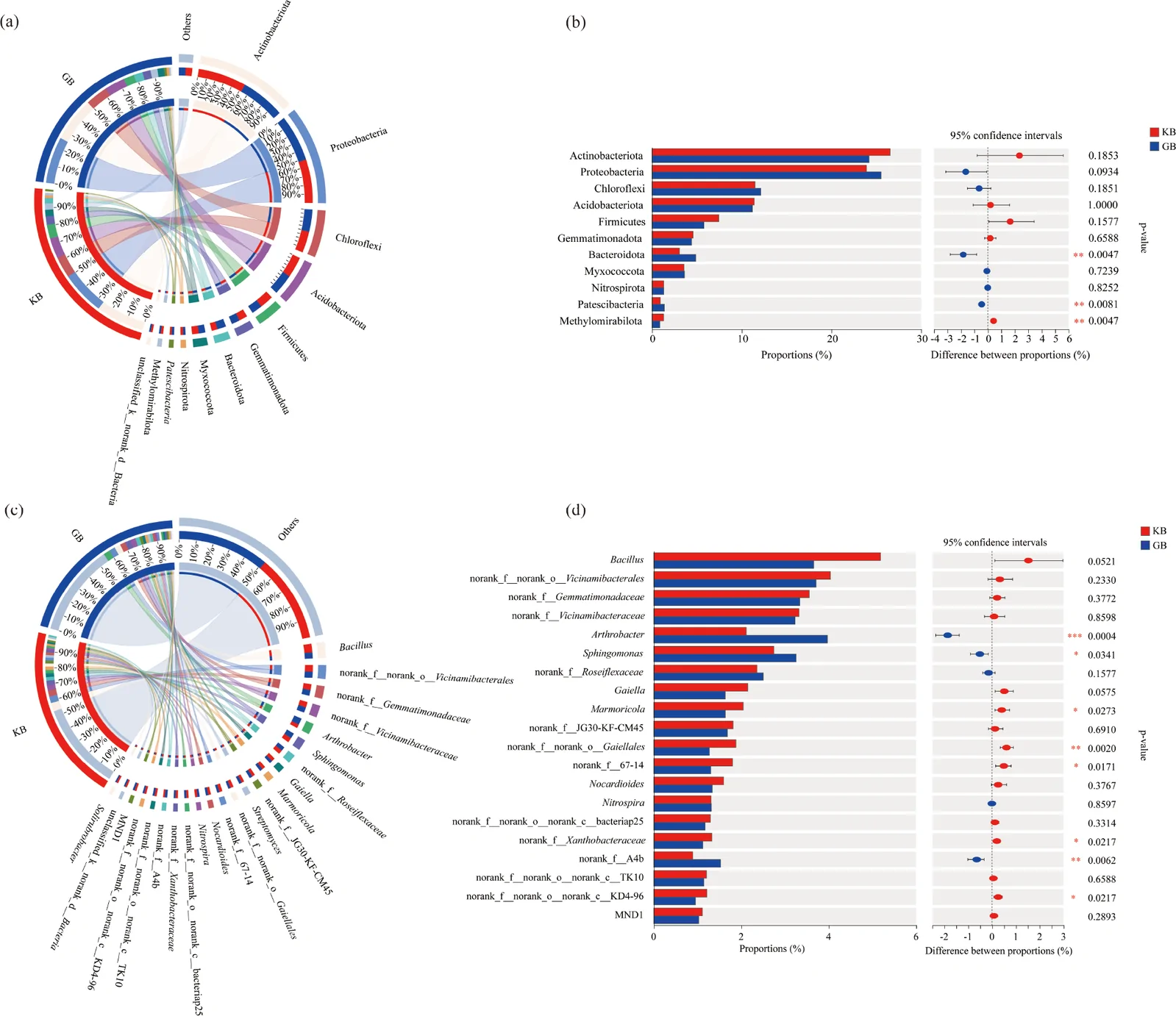
Proportions (**a**) and Wilcoxon rank-sum test (**b**) at the phylum level, and proportions (**c**) and Wilcoxon rank-sum test (**d**) at the genus level of rhizosphere bacteria between KB and GB watermelon cultivar groups.

At the bacterial phylum level, the rhizosphere of *Fusarium* wilt-resistant watermelon cultivar group (KB) and susceptible watermelon cultivar group (GB) harbored 11 and 10 dominant bacterial phyla (relative abundances > 1%), respectively (Fig. 2a). Among these, the relative abundances of Actinobacteriota (KB: 26.54%; GB: 24.19%), Acidobacteriota (KB: 11.4%; GB: 11.2%), Firmicutes (KB: 7.45%; GB: 5.80%), and Gemmatimonadota (KB: 4.60%; GB: 4.42%) were descriptively higher in KB, though these differences were not statistically significant. Additionally, Methylomirabilota (1.31%) was uniquely identified as a dominant phylum only in KB. Conversely, Kruskal–Wallis rank-sum tests revealed that the relative abundances of Bacteroidota and Patescibacteria were significantly higher in GB than in KB (Fig. 2b).

At the bacterial genus level, KB and GB cultivar groups possessed 22 and 20 dominant bacterial genera (relative abundances > 1%), respectively (Fig. 2c). Genera with higher relative abundances in KB included *Bacillus* (KB: 5.19%; GB: 3.66%), *Marmoricola* (KB: 2.05%, 1.64%), *norank_f__norank_o__Gaiellales* (KB: 1.88%; GB: 1.27%), *norank_f__67-14* (KB: 1.80%; GB: 1.31%), and *orank_f__Xanthobacteraceae* (KB: 1.33%; GB: 1.13%; Fig. 2d). KB also harbored several unique dominant genera such as *norank_f__Vicinamibacteraceae* (3.32%), *norank_f__Roseiflexaceae* (2.86%), *norank_f__norank_o__norank_c__KD4-96* (1.22%), and *Solirubrobacter* (1.02%). In contrast, the relative abundances of *Arthrobacter* (GB: 3.97%; KB: 2.12%) and *Sphingomonas* (GB: 3.26%; KB: 2.75%) were significantly higher in GB than in KB. These findings indicate that the dominant bacterial phyla and genera differed between resistant and susceptible watermelon cultivars, exhibiting both significant abundance variations and distinct taxonomic compositions.

**Fig 3:**
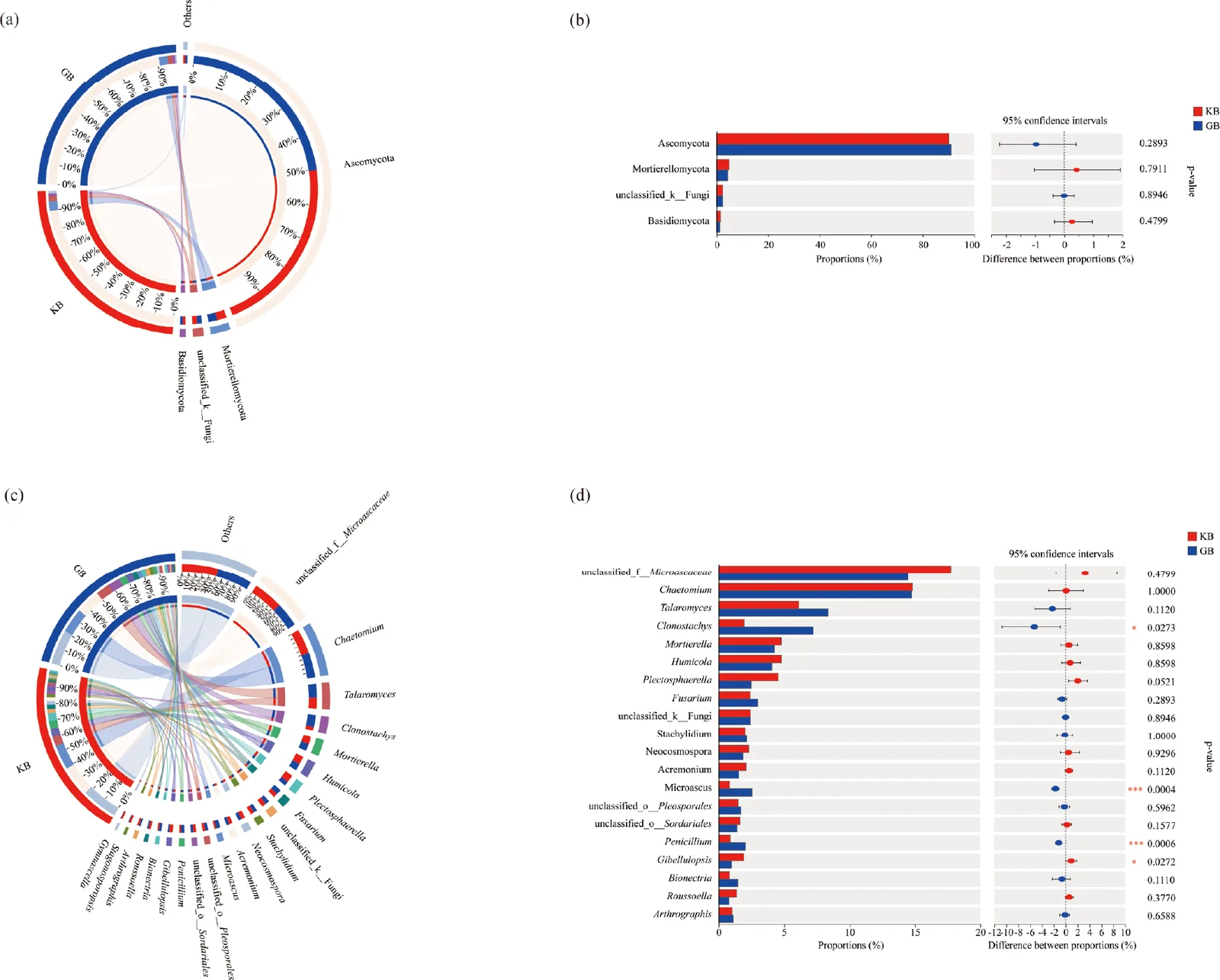
Proportions (**a**) and Wilcoxon rank-sum test (**b**) at the phylum level, and proportions (**c**) and Wilcoxon rank-sum test (**d**) at the genus level of rhizosphere fungi between KB and GB watermelon cultivar groups.

At the fungal phylum level, both *Fusarium* wilt-resistant (KB) and -susceptible watermelon (GB) cultivar groups harbored the same four dominant fungal phyla (relative abundances >1%) in the rhizosphere (Fig. 3a): namely Ascomycota (KB: 90.06%; GB: 91.02%), Mortierellomycota (KB: 4.83%; GB: 4.40%), unclassified_k__Fungi (KB: 2.43%; GB: 2.44%), and Basidiomycota (KB: 1.54%; GB: 1.27%). Kruskal–Wallis rank-sum tests showed no significant differences (*P* > 0.05) in relative abundances of these fungal phyla between KB and GB (Fig. 3b), with Ascomycota being the most dominant phylum in both cultivars.

At the fungal genus level, KB and GB rhizospheres contained 19 and 18 dominant genera, respectively (Fig. 3c). The relative abundances of *unclassified_f__Microascaceae* (KB: 17.75%; GB: 14.47%) and *Plectosphaerella* (KB: 4.54%; GB: 2.50%) were higher in KB. In contrast, the relative abundance of *Clonostachys* (KB: 1.95%; GB: 7.22%) was significantly higher in GB than in KB (*P* < 0.05). Additionally, *Gibellulopsis* (1.92%) was identified as the unique dominant genus in KB, while *Microascus* (2.55%) and *Penicillium* (2.06%) were uniquely dominant in GB. These findings suggest that differences between *Fusarium* wilt-resistant and -susceptible watermelon cultivars are not reflected in broad shifts at the phylum level but are instead associated with variations in the relative abundance of differentially abundant genera.

**Fig 4:**
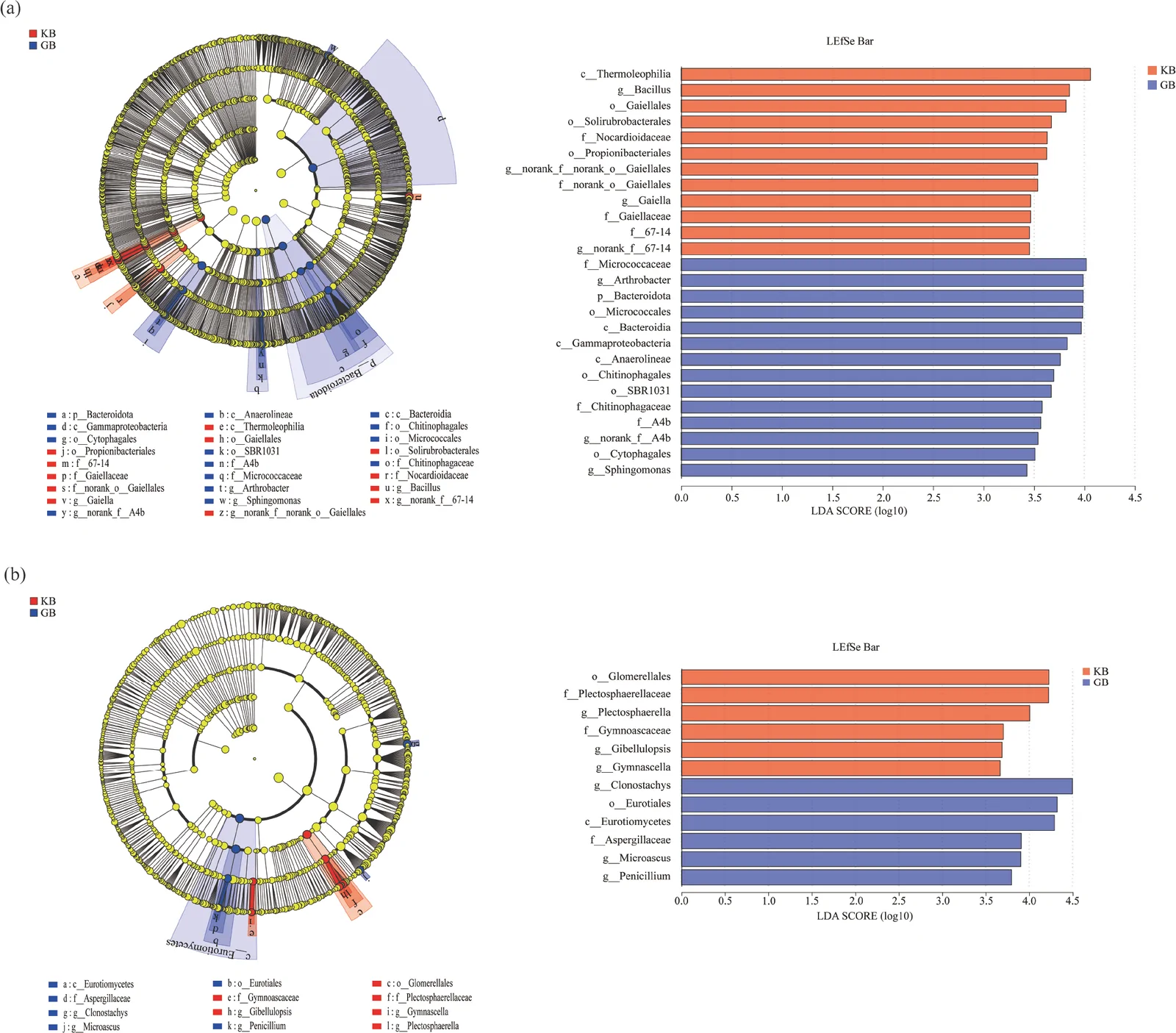
LEfSe analysis of rhizosphere bacteria (**a**) and fungi (**b**) between KB and GB watermelon cultivar groups. Nodes of different colors indicate microbial groups that are significantly enriched in the corresponding groups and have a significant impact on the differences between groups; light yellow nodes represent microbial groups that show no significant differences in different groups or have no significant influence on the differences between groups.

To identify key microbial taxa associated with *Fusarium* wilt resistance, LEfSe analysis was conducted on the rhizosphere microbiomes of resistant and susceptible watermelon cultivar groups. A total of 26 bacterial taxa exhibited significant differences (*P* < 0.05, LDA score > 3.4; Fig. 4). At the genus level, features with higher relative abundance in KB included *Bacillus*, *norank_f__norank_o__Gaiellales*, *Gaiella*, and *norank_f__67-14*. Features with higher relative abundance in GB included *Arthrobacter* and *norank_f__A4b* (Fig. 4a).

LEfSe analysis of rhizosphere fungi identified 12 significant differentially fungal taxa (*P* < 0.05, LDA score > 3.5). Features with higher relative abundance in KB included *Plectosphaerella*, *Gibellulopsis*, and *Gymnascella*. Features with higher relative abundance in GB included *Clonostachys*, *Microascus*, and *Penicillium* (Fig. 4b). LEfSe analysis pinpointed distinct rhizosphere microbiome differentiation between *Fusarium* wilt-resistant and -susceptible watermelon cultivar groups at the genus level, identifying several key taxa significantly enriched in each group.

### Co-occurrence network structure of rhizosphere soil microbiota in *Fusarium* wilt-resistant and -susceptible watermelon cultivar groups

To investigate the interspecies relationships of rhizosphere soil microorganisms in watermelon cultivar groups with differing resistance levels, a univariate correlation network analysis was performed (Spearman correlation coefficient ≥0.5, *P* < 0.05).

**Fig 5:**
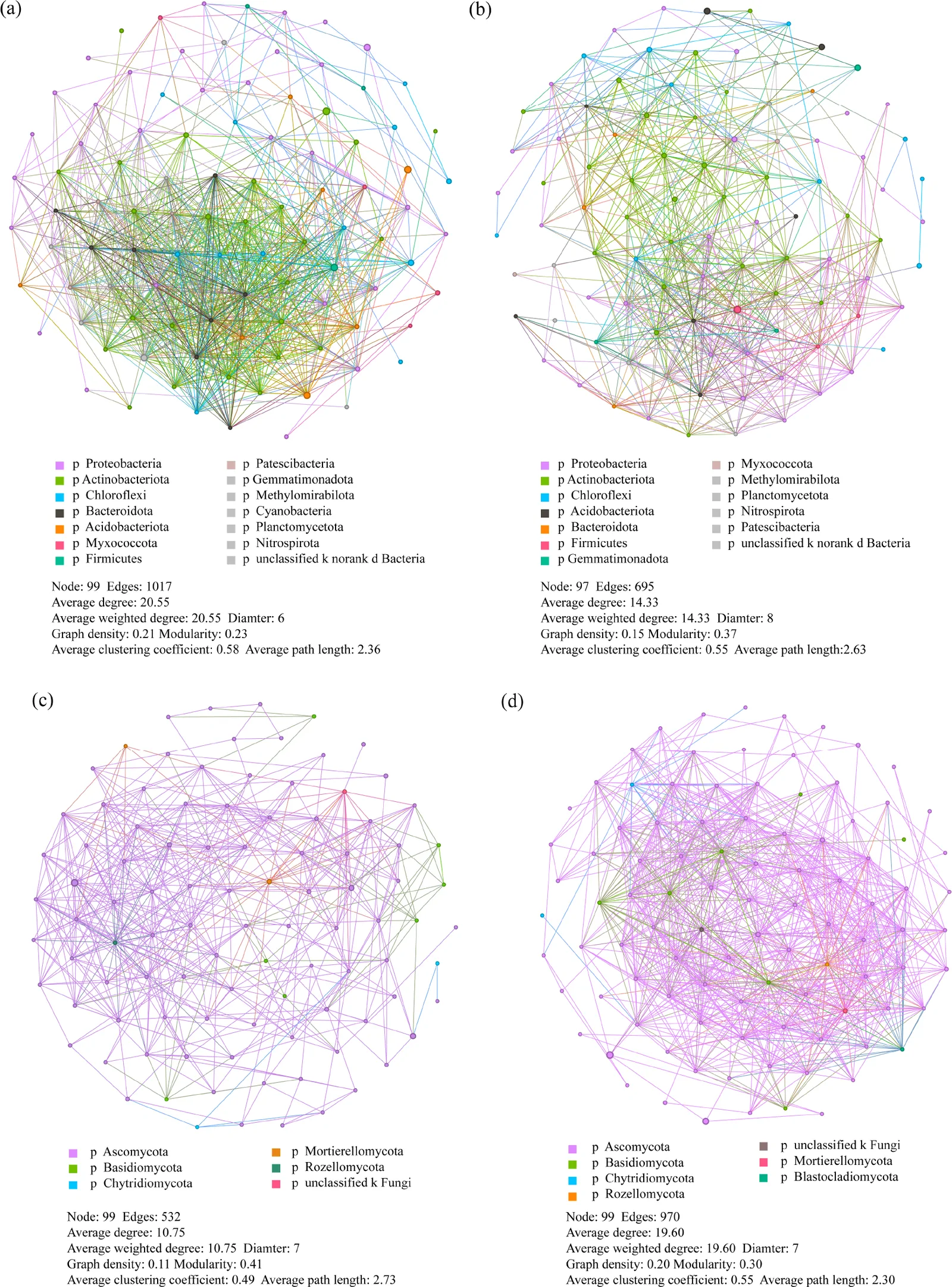
Co-occurrence network of the bacteria in GB (**a**) and KB (**b**); co-occurrence network of the fungi in GB (**c**) and KB (**d**); each node represents a single genus, and different colors represent different phyla.

The results revealed that the bacterial network of the resistant watermelon cultivar group (KB) had fewer nodes (97) and edges (695; Fig. 5b) than those of the susceptible (GB), which contained 99 nodes and 1,017 edges (Fig. 5a). Additionally, KB exhibited a lower average degree (14.33) and graph density (0.15) than those of GB (average degree: 20.55; graph density: 0.21). However, KB had a greater diameter (8), modularity (0.37), and average path length (2.63) compared to GB (diameter: 6; modularity: 0.23; average path length: 2.36). The bacterial network in the susceptible cultivar group (GB) exhibited higher connectivity. In contrast, the network in the resistant cultivar group (KB) was sparser but possessed significantly greater modularity and a longer average path length.

The fungal network of KB demonstrated equal or higher structural complexity relative to GB, with an identical number of nodes (99) but substantially more edges (970), a higher average degree (19.60), diameter (7), graph density (0.20), and average clustering coefficient (0.55; Fig. 5d). These values surpassed or matched those of GB’s fungal network (edges: 532; average degree: 10.75; diameter: 7; graph density: 0.11; average clustering coefficient: 0.49; Fig. 5c). While the fungal networks of both cultivars contained the same number of nodes, the resistant cultivar group (KB) possessed nearly twice as many edges, along with higher average degree, network density, and clustering coefficient. These topological variations reveal that the rhizosphere KB hosts a more highly connected co-occurrence network, distinguished by denser associations among fungal taxa and an increased propensity for taxa to form clustered, interconnected relationships.

### Functional prediction of soil microbiota in rhizosphere between *Fusarium* wilt-resistant and -susceptible watermelon cultivar groups

**Fig 6:**
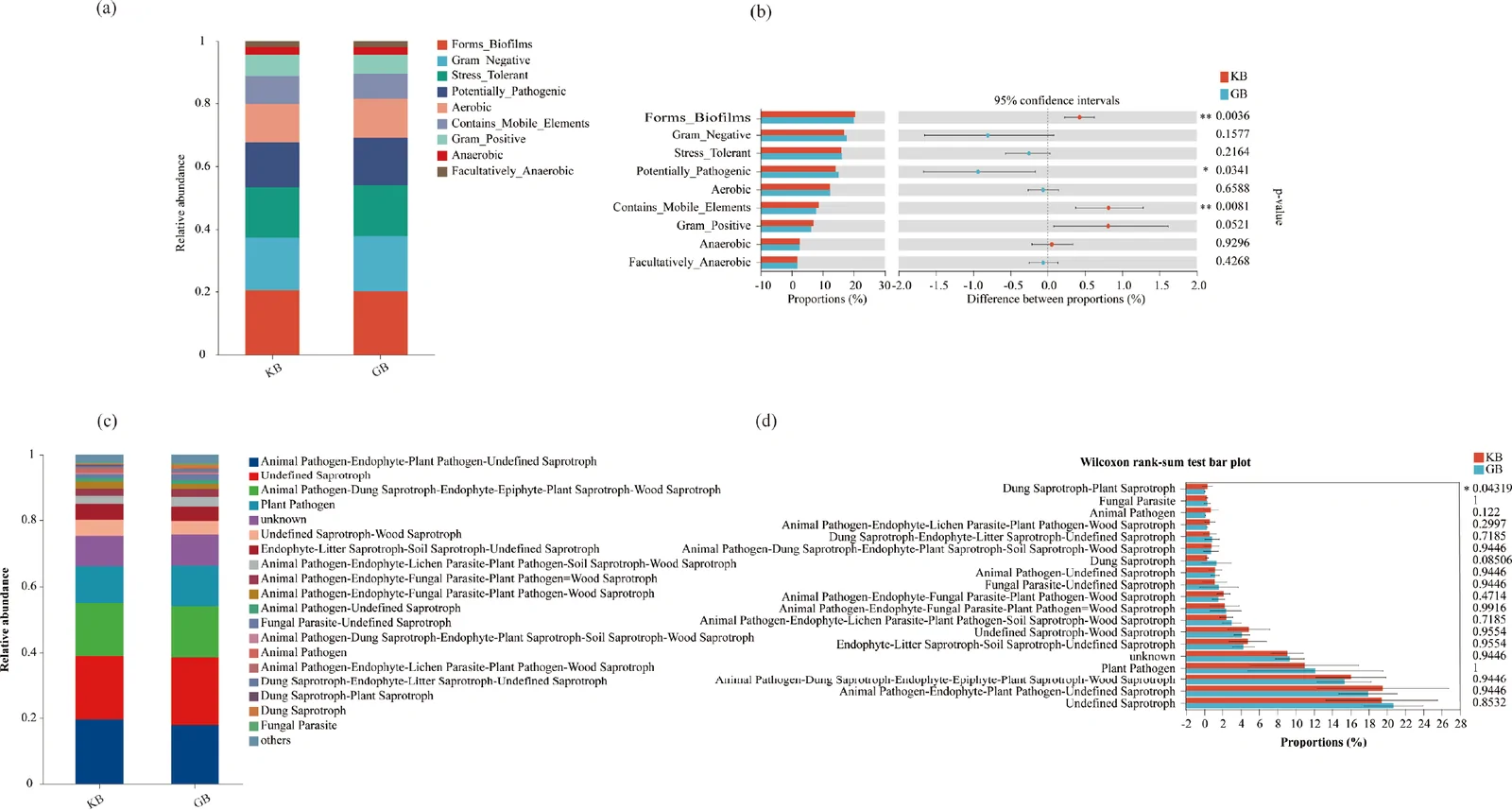
Prediction (**a**) and Wilcoxon rank-sum test (**b**) of BugBase phenotypes of rhizosphere bacteria between KB and GB watermelon cultivar groups. (**c**) Relative abundance and Wilcoxon rank-sum test (**d**) of fungal functional groups (guilds) based on OTU annotation table with distribution frequency levels of KB and GB watermelon cultivar groups. Note: *0.01 < *P* ≤ 0.05 and **0.001 < *P* ≤ 0.01.

BugBase analysis was performed on the rhizosphere bacterial communities of resistant and susceptible cultivar groups to assess their predicted functional phenotypes in relation to disease resistance. The results showed that the predominant bacterial phenotypes in both resistant and susceptible cultivar groups were Forms_Biofilms, Gram_Negative, Stress_Tolerant, Potentially_Pathogenic, Aerobic, Contains_Mobile_Elements, Gram_Positive, Anaerobic, and Facultatively_Anaerobic (Fig. 6a). Among these, the relative abundance of Forms_Biofilms and Contains_Mobile_Elements was significantly higher in KB, while Potentially_Pathogenic was significantly higher in GB (*P* < 0.05; Fig. 6b).

FUNGuild functional prediction of rhizosphere fungi in resistant and susceptible watermelon cultivar groups identified 19 ecological guilds (Fig. 6c). Although most functional groups did not reach statistical significance, the rhizosphere of the resistant cultivar group (KB) descriptively exhibited higher relative abundances of several saprotrophic and endophytic fungal guilds, such as Animal Pathogen–Endophyte–Plant Pathogen–Undefined Saprotroph, Animal Pathogen–Dung Saprotroph–Endophyte–Epiphyte–Plant Saprotroph–Wood Saprotroph, Endophyte–Litter Saprotroph–Soil Saprotroph–Undefined Saprotroph, Undefined Saprotroph–Wood Saprotroph, Animal Pathogen–Endophyte–Fungal Parasite–Plant Pathogen–Wood Saprotroph, and Animal Pathogen–Undefined Saprotroph. Notably, the functional guild Dung Saprotroph–Plant Saprotroph was significantly more abundant in KB than in the susceptible cultivar group (GB; *P* < 0.05; Fig. 6d), suggesting that these functional groups may directly enhance plant immunity against pathogens, potentially through the induction of systemic resistance.

## DISCUSSION

In recent years, advances in plant–microbe interaction research have demonstrated that plants can actively shape their rhizosphere microbiomes (20), and different genotypes or cultivars recruit distinct microbial communities even under identical soil conditions (21). In the present study, the resistant and susceptible treatment groups each comprised multiple watermelon cultivars, which are hereafter designated as the resistant (KB) and susceptible (GB) cultivar groups. By comparing *Fusarium* wilt-resistant and -susceptible watermelon cultivar groups, we further validated this concept. We found that:

Significant differences exist in the fungal richness and taxonomic composition of rhizosphere microbiomes between watermelon cultivar groups with contrasting resistance levels. The resistant cultivar group not only exhibits higher fungal richness and a greater number of unique OTUs compared with the susceptible cultivar group but also shows higher relative abundances of several differentially abundant taxa, including *Bacillus*, *Gaiella*, *Solirubrobacter*, *Gibellulopsis*, and *Gymnascella*. Studies have demonstrated that *Bacillus* benefits plants through mechanisms like nitrogen fixation, phosphate solubilization, siderophore production, synthesis of plant growth hormones, and suppression of plant pathogens (22). *Gaiella* plays roles in nitrogen cycling, carbon metabolism, phosphorus uptake, and organic matter decomposition (23, 24). *Solirubrobacter* has shown potential for plant–microbe interactions and the biosynthesis of bioactive compounds (25). *Gibellulopsis*, known as an endophytic fungus, has been shown to suppress Verticillium wilt and induce defense responses in cotton plants (26). Fungi belonging to the genus *Gymnascella* are capable of producing large quantities of volatile and non-volatile compounds with potent antifungal and anticancer activity, exhibiting high inhibitory efficacy against *Fusarium solani* and *Alternaria alternata* (27). Importantly, plant resistance is not determined by the presence of individual beneficial taxa alone, but rather by the overall structure, diversity, and functional integration of the rhizosphere microbiome. The rhizosphere microbiome is increasingly acknowledged as a key extension of the plant immune system, where the combined functional activities of resident microbes exert a pivotal role in mediating disease suppression (1, 28). In this context, the higher diversity and enriched functional taxa observed in the resistant cultivar group may contribute to a more stable and resilient microbial network. While *Clonostachys* is widely recognized for its antagonistic activity against plant pathogens, its enrichment alone may not be sufficient to confer effective disease resistance. This may indicate that, although susceptible cultivar groups can recruit beneficial microorganisms, resistant cultivars harbor a more diverse microbial community. Similar patterns have been reported in tomato and disease-suppressive soil systems, where plant resistance was associated with complex beneficial microbial assemblages and enhanced community interactions rather than the enrichment of individual antagonistic taxa alone (29–31). Such community structures are likely more susceptible to pathogen invasion, as reduced microbial diversity and network instability have been closely associated with rhizosphere dysbiosis and compromised plant health (29, 32). Consistent with these findings, elevated fungal diversity and enrichment of keystone taxa in resistant cultivars have been documented in melon and other plant species, implying that the assembly of a functionally redundant and modular microbiome may constitute a conserved strategy for enhancing fungal disease resistance (33, 34). Taken together, these findings indicate that both resistant and susceptible cultivar groups possess microbiome-mediated defense mechanisms; however, the effectiveness of these mechanisms may depend on microbial diversity, network organization, and cooperative interactions within the community. The enrichment of a narrow range of antagonistic taxa may be associated with reduced microbial diversity and functional redundancy, which have been linked to community instability and rhizosphere dysbiosis in previous studies (32, 35). In contrast, resistant cultivars (KB) were characterized by the enrichment of taxa such as unclassified_f__*Microascaceae* and *Plectosphaerella*. Collectively, these findings indicate that watermelon resistance may be associated with specific assembly patterns of rhizosphere fungal communities: resistant cultivar groups were characterized by higher fungal richness and more complex community organization, whereas susceptible cultivar groups appeared to be associated with the enrichment of specific antagonistic taxa. However, these patterns should be interpreted within the broader framework of plant holobiont complexity, where disease resistance is shaped by dynamic interactions among host immune responses, resident microbial communities, and fluctuating environmental factors (36–38). As such, the observed associations are more likely to reflect potential ecological relationships rather than definitive causal mechanisms.

The taxa enriched in the rhizosphere of resistant cultivar groups and the associated co-occurrence networks exhibit distinct topological characteristics compared with those of susceptible cultivar groups. In bacterial networks, susceptible cultivar groups display denser and more frequent interactions among bacterial taxa. However, increased connectivity does not inherently signify greater ecological stability, as recent studies have demonstrated that highly connected microbial communities may also be more sensitive to external disturbances, depending on interaction types, the balance of positive and negative links, and overall network organization (28, 39). In contrast, the highly modular structure of resistant cultivar groups indicates that bacterial communities are organized into several functionally semi-independent yet internally cohesive subunits. Such modular organization is thought to buffer the spread of disturbances within ecological networks by confining perturbations to localized compartments, thereby contributing to community persistence (40, 41). Similarly, reference 42 reported that more complex and stable soil microbial interaction networks were associated with improved plant health and reduced disease incidence, highlighting the ecological importance of network organization in plant–microbiome systems. In the context of this study, such disturbances may include pathogen invasion or other environmental stresses. When challenged by pathogen invasion or other stresses, modular network organization may help confine disturbances within localized compartments rather than allowing them to spread throughout the entire network. Such characteristics have been associated with enhanced community robustness in previous studies, although their ecological significance in watermelon *Fusarium* wilt remains to be validated. The fungal network of the resistant cultivar group (KB) contained nearly twice as many edges as that of the susceptible cultivar group (GB), with significantly higher average degree and clustering coefficient, reflecting more complex and tightly coordinated interactions. Rather than interpreting connectivity alone as inherently beneficial or detrimental, these results suggest that bacterial and fungal communities may differ fundamentally in their network organization and interaction patterns. Therefore, network connectivity should be interpreted in conjunction with other topological properties, such as modularity, clustering coefficient, and overall network structure, rather than as an isolated indicator of ecological stability or robustness. In the fungal network, the combination of greater connectivity and higher clustering coefficient may indicate tighter local associations and enhanced functional redundancy, which have been linked to improved ecosystem functioning and microbiome robustness in recent studies (28, 41). It should be noted that co-occurrence network analyses are based solely on correlational relationships and do not directly measure ecological processes such as stability, resilience, or functional coordination. Therefore, the interpretations presented here reflect potential ecological implications rather than experimentally validated mechanisms. Future studies incorporating pathogen challenge experiments and temporal monitoring of microbial community dynamics will be necessary to validate these hypotheses and determine how microbial networks respond to pathogen invasion. Such highly modular and redundant network structures may contribute to the maintenance of community functioning and could potentially restrict pathogen colonization through mechanisms including resource competition, spatial occupation, or the production of antagonistic metabolites (41).

At the community level, functional potential and structural characteristics exhibit associated patterns that can partially explain differences in resistance phenotypes. BugBase predictions indicated that the bacterial community of the resistant cultivar group was significantly enriched in the phenotypes Forms_Biofilms and Contains_Mobile_Elements. Forms_Biofilms represents the ability to form biofilms, which facilitates the colonization of beneficial microbes in the rhizosphere and the establishment of stable microcolonies, thereby enabling more effective niche occupation and protection against pathogen invasion (43). Contains_Mobile_Elements refers to the major carriers of resistance genes and virulence factors, which may be involved in the horizontal transfer of antibiotic resistance genes and secondary metabolite biosynthetic genes, thus promoting the collective evolution of microbial defense capacity. In contrast, the susceptible cultivar group was enriched in Potentially_Pathogenic taxa, suggesting that its microecological environment may be more favorable for the proliferation of harmful microorganisms, thereby increasing the risk of disease. FUNGuild analysis further revealed that the resistant cultivar group was significantly enriched in the functional guild Dung Saprotroph–Plant Saprotroph. These fungi are capable of decomposing complex organic matter (44, 45), and their enhanced activity may indicate a more active nutrient cycling process in the KB rhizosphere. This not only promotes healthy plant growth but may also generate metabolites during decomposition that directly suppress soilborne pathogens. Such functional traits not only reflect the ecological roles of the community but may also directly enhance plant immunity by inducing host systemic resistance or facilitating the dissemination of resistance genes.

Based on the above results, we found that the differential characteristics and potential functions of fungal communities were more pronounced than those of bacterial communities in watermelon cultivars with contrasting resistance to *Fusarium* wilt. This observation is consistent with Qiu et al.’s (33) finding of greater fungal richness in *Fusarium* wilt-resistant melon cultivars, but contrasts with the conclusion of Xia et al. (46), who reported that bacterial responses predominated in tobacco. Such differences may be attributable to the distinct nature of the pathogens involved, as *Fusarium* wilt in watermelon and melon is caused by fungi, whereas the wilt disease in tobacco is caused by bacteria. Finally, it is important to acknowledge that our analysis focuses on baseline rhizosphere microbial communities. Given that microbial composition can undergo dynamical shifts during pathogen infection, taxa recruited post-infection may play a critical role in plant defense responses. Future research should therefore investigate the temporal dynamics of rhizosphere microbiomes under disease pressure to better understand these adaptive changes. In summary, this study links structural characteristics with functional potential, revealing differences in rhizosphere microbial community composition between resistant and susceptible watermelon cultivar groups. This comparative analysis also provides a perspective within core plant microbiome frameworks, including dysbiosis, pathobiome, and the cry-for-help hypothesis, to better interpret microbiome-based resistance. This comparative analysis not only deepens our understanding of plant–microbe interactions but also provides new perspectives for elucidating the microbiological mechanisms underlying plant disease resistance. It should be noted that the functional predictions presented in this study, based on amplicon sequencing data (16S rRNA gene and ITS), are inferential in nature and do not directly represent measured microbial activities. Although these predictions provide useful insights into potential functional shifts, they should be interpreted with caution. Future studies integrating metatranscriptomic, metabolomic, or culture-dependent approaches will be required to validate these functional hypotheses and better elucidate the mechanistic roles of rhizosphere microbiomes in disease resistance.

### Conclusion

In conclusion, watermelon cultivars with varying levels of disease resistance exhibit distinct rhizosphere microbial communities in terms of taxonomic composition, diversity, and co-occurrence network structure. Resistant cultivars (KB) harbored a greater abundance of unique microbial taxa and a more highly connected fungal network, whereas susceptible cultivars (GB) were characterized by the enrichment of specific dominant taxa. Functional predictions revealed significant differences in traits such as biofilm formation and the presence of mobile genetic elements, underscoring the critical role of host genotype in shaping both the structural and functional potential of the rhizosphere microbiome. These baseline community patterns suggest a potential link between microbiome organization and disease resistance, which can be interpreted within established ecological frameworks, including dysbiosis, pathobiome dynamic, and the cry-for-help hypothesis. It is important to note that microbial communities may undergo further dynamic shifts during pathogen infection, a temporal dimension that warrants investigation in subsequent research.

This study provides a comprehensive ecological characterization of rhizosphere microbiomes in watermelon cultivars with contrasting disease resistance phenotypes, establishing a foundational framework for future investigations into the functional links between microbial community attributes and plant health outcomes. More broadly, our findings contribute to an improved understanding of genotype-associated variation in plant–microbiome systems and offer a valuable reference for future research aimed at developing microbiome-based strategies for sustainable crop disease management.

## Data Availability

The data presented in this study are openly available in National Center for Biotechnology Information at https://www.ncbi.nlm.nih.gov/, accessed on 10 August 2025, reference numbers PRJNA1304008, PRJNA1304011.
